# Supplementary material for: Prospective identification of functionally distinct stem cells and neurosphere-initiating cells in adult mouse forebrain
Source: eLife. 2014 May 7;3:e02669. doi: 10.7554/eLife.02669 (PMC4038845; doi:10.7554/eLife.02669)
Supplement: Supplementary file 1. — (A) List of antibody markers screened to purify neurosphere-initiating cells. (B) List of growth factors screened to stimulate pre-GEPCOT growth. DOI: http://dx.doi.org/10.7554/eLife.02669.015 [file elife02669s001.docx]

**SUPPLEMENTARY TABLES**

| **Supplementary File 1A: Candidate markers used for screening for neurosphere-initiating cell markers.** | | | | |
| --- | --- | --- | --- | --- |
| **Antigen** | **Antibody source** | **All SVZ cells?** | **Enriches neurosphere-initiating cells?** | **GEPCOT cells?** |
| CD45 cl. 30-F11 | Biolegend | Heterogeneous | Yes | - |
| Ter119 | Biolegend | Heterogeneous | Yes | - |
| CD24 cl. M1/69 | eBioscience | Heterogeneous | Yes | -/low |
| O4 | Morrison lab | Heterogeneous | Yes | -/low |
| PSA-NCAM cl. 5A5 | DSHB | Heterogeneous | Yes | -/low |
| CD200 cl. OX-90 | BD Bioscience | Heterogeneous | Yes | Heterogeneous but neurosphere-initiating cells are enriched in -/low fraction |
| 8.1.1 | DSHB | Heterogeneous | Yes | Heterogeneous but neurosphere-initiating cells are enriched in high fraction |
| CD172a cl. P84 | BD Bioscience | Heterogeneous | Yes | Heterogeneous but neurosphere-initiating cells are enriched in -/low fraction |
| EGFR cat. BAF1280 | R&D Systems | Heterogeneous | Yes | High |
| PlexinB2 cl. 3E7 | eBioscience | Heterogeneous | Yes | High |
| Glast cl. ACSA-1 | Miltenyi | Heterogeneous | Yes | Mid |
| HNK1/CD57 cl. VC1.1 | Sigma | Heterogeneous | Yes | -/low |
| CD81 cl. Eat-2 | eBioscience | Heterogeneous | Yes | High |
| CD56 cl. 13 | BD Bioscience | Heterogeneous | Yes | -/low |
| LTA lectin | EY Labs | Heterogeneous | Yes | -/low |
| PHA-L lectin | EY Labs | Heterogeneous | Yes | -/low |
| 647-Egf | Life Technologies | Heterogeneous | Yes | High |
| FORSE-1 | DSHB | Heterogeneous | No | Heterogeneous |
| 4D4 (A2B5-like) | DSHB | Heterogeneous | No | Heterogeneous |
| CD106/VCAM1 cl. MK2 | Cedarlane | Heterogeneous | No | Heterogeneous |
| Thy1-1 cl. OX7 | eBioscience | Heterogeneous | No | Heterogeneous |
| OCAM/NCAM2 cat. AF778 | R&D Systems | Heterogeneous | No | Heterogeneous |
| CD166 cl. ALC48 | eBioscience | Heterogeneous | No | Heterogeneous |
| CD98 cl. RL388 | eBioscience | Heterogeneous | No | Homogeneous |
| S1P1/EDG-1 cl. 713412 | R&D Systems | Heterogeneous | No | Homogeneous weak |
| SNA-2 lectin | EY Labs | Heterogeneous | No | No staining |
| CD53 cl. OX-79 | BD Bioscience | Heterogeneous | No | No staining |
| PHA-E lectin | EY Labs | Heterogeneous | No | No staining |
| MPA lectin | EY Labs | Heterogeneous | No | No staining |
| CD321 cl. H202-106 | AbD Serotec | Heterogeneous | No | No staining |
| Ly24 cl. 5D2-27 | DSHB | Heterogeneous | No | No staining |
| CD49e cl. HMa5-1 | eBioscience | Heterogeneous | No | No staining |
| CD71 cl. RI7 217.1.4 | eBioscience | Heterogeneous | No | No staining |
| ConA lectin | EY Labs | Heterogeneous | No | Unknown |
| LcH lectin | EY Labs | Heterogeneous | No | Unknown |
| GNA lectin | EY Labs | Heterogeneous | No | Unknown |
| PSA lectin | EY Labs | Heterogeneous | No | Unknown |
| WFA lectin | EY Labs | Heterogeneous | No | Unknown |
| DBA lectin | EY Labs | Heterogeneous | No | Unknown |
| Pokeweed lectin | EY Labs | Heterogeneous | No | Unknown |
| A2B5 cl. A2B5-105 | Millipore | Heterogeneous | No | Heterogeneous but use clone 4D4 instead |
| CD106/VCAM1 cat. AF643 | R&D Systems | Heterogeneous | No | Heterogeneous but use clone MK2 instead |
| DSL lectin | Vector Labs | Heterogeneous | No | Heterogeneous but use STL instead |
| LEL lectin | Vector Labs | Heterogeneous | No | Heterogeneous but use STL instead |
| CD15 cl. SSEA-1 | DSHB | Heterogeneous | Limited | Heterogeneous |
| EMA-1 | DSHB | Heterogeneous | Limited | Heterogeneous |
| STL lectin | Vector Labs | Heterogeneous | Limited | Heterogeneous |
| CD15 cl. MMA | BD Bioscience | Heterogeneous | Limited | Heterogeneous but use clone SSEA1 instead |
| WGA lectin | EY Labs | Heterogeneous | Limited | Heterogeneous but use STL instead |
| CD9 cl. KMC8 | eBioscience | Uniformly positive | -- | Uniformly positive |
| SynCAM4 cl. N244/5 | NeuroMab | Uniformly positive | -- | Uniformly positive |
| CD98 cl. H202-41 | BD Bioscience | Uniformly positive | -- | Uniformly positive |
| SNA-1 lectin | EY Labs | Uniformly positive | -- | Uniformly positive |
| CDH19 cat. H00028513-B01P | Abnova | Weak staining | -- | -- |
| CD184/CXCR4 cl. 2B11 | eBioscience | Weak staining | -- | -- |
| c-Kit cat. BAF1356 | R&D Systems | Weak staining | -- | -- |
| c-Kit cl. 2B8 | eBioscience | Weak staining | -- | -- |
| CD51 cl RMV-7 | eBioscience | Weak staining | -- | -- |
| CSPG5 cat. AF5665 | R&D Systems | Weak staining | -- | -- |
| CD316/IGSF8 AF3117 | R&D Systems | Weak staining | -- | -- |
| p75 cl. AB1554 | Millipore | Weak staining | -- | -- |
| CD49f cl. GoH3 | eBioscience | Weak staining | -- | -- |
| p75 cl. MAB5744 | Millipore | Weak staining | -- | -- |
| MHC-I cl. SF1-1.1.1 | eBioscience | Weak staining | -- | -- |
| CSPG cl.CS-56 | Sigma | Weak staining | -- | -- |
| CD47 cl. miap301 | BD Bioscience | Weak staining | -- | -- |
| c-Met cl. eBioclone7 | eBioscience | Weak staining | -- | -- |
| vRobo1 | DSHB | Weak staining | -- | -- |
| VVA lectin | EY Labs | Weak staining | -- | -- |
| c-Kit cl. ACK2 | eBioscience | Weak staining | -- | -- |
| CD140a/PDGFRa cl. APA5 | eBioscience | Weak staining | -- | -- |
| CD133 cl. 13A4 | eBioscience | Weak staining | -- | -- |
| CD29 cl. Ha2/5 | BD Bioscience | Weak staining | -- | -- |
| CD29 cl. HMB1-1 | eBioscience | Weak staining | -- | -- |
| LBA lectin | EY Labs | Weak staining | -- | -- |
| BPA lectin | EY Labs | Weak staining | -- | -- |
| ACA lectin | EY Labs | Weak staining | -- | -- |
| Lotus lectin | EY Labs | Weak staining | -- | -- |
| CD64a,b cl. X54-5/7.1 | BD Bioscience | Weak staining | -- | -- |
| CD202b cl. TEK4 | eBioscience | Weak staining | -- | -- |
| CD31 cl. 390 | eBioscience | Weak staining | -- | -- |
| CD3 cl. 17A2 | eBioscience | Weak staining | -- | -- |
| AIA lectin | EY Labs | Weak staining | -- | -- |
| 40E-C | DSHB | Weak staining | -- | -- |
| Notch1 cl. HMN1-12 | Biolegend | Weak staining | -- | -- |
| CD162 cl. 2PH1 | BD Bioscience | Weak staining | -- | -- |
| Gpc1 cat. AF4519 | R&D Systems | Weak staining | -- | -- |
| Traf6 cat. 1660-1 | Epitomics | Weak staining | -- | -- |
| NG2 cat. AB5320 | Millipore | Weak staining | -- | -- |
| Lepr cat. BAF497 | R&D Systems | Weak staining | -- | -- |
| TrkB cat. Ab7219 | Abcam | Weak staining | -- | -- |
| BGN | unknown | Weak staining | -- | -- |
| CD55 cl. RIKO-3 | Biolegend | Weak staining | -- | -- |
| NK1.1 cl. PK136 | eBioscience | Weak staining | -- | -- |
| Fitc-bFGF | Morrison lab | Weak staining | -- | -- |
| Sca-1 cl. D7 | eBioscience | Weak staining | -- | -- |
| Syndecan-4 cl. KY/8.2 | BD Bioscience | Weak staining | -- | -- |
| CD107a cl. 1D4B | eBioscience | Weak staining | -- | -- |
| B30 | DSHB | Weak staining | -- | -- |
| CD182 cl. TG11/CXCR2 | Biolegend | Weak staining | -- | -- |
| CD197 cl. 4B12 | BD Bioscience | Weak staining | -- | -- |
| hFLRT1 cat. AF2794 | R&D Systems | Weak staining | -- | -- |
| CD63 cl. AD1 | BD Bioscience | Weak staining | -- | -- |
| ADAM23 cl. GW21429 | Sigma | Weak staining | -- | -- |
| CD100 cl. BMA12 | eBioscience | Weak staining | -- | -- |
| hFLRT2 cl. AF2877 | R&D Systems | Weak staining | -- | -- |
| LtbR cl. 3C8 | eBioscience | Weak staining | -- | -- |
| Gfra1 cat. AF560 | R&D Systems | Weak staining | -- | -- |
| Robo2 cat. AF3147 | R&D Systems | Weak staining | -- | -- |
| TrkC cat. ab51190 | Abcam | Weak staining | -- | -- |
| Endoglycan cl. AF3534 | R&D Systems | Weak staining | -- | -- |
| rRobo1 cat. AF1749 | R&D Systems | Weak staining | -- | -- |
| OPCML cat. NBP1-41484 | Novus | Weak staining | -- | -- |
| TrkB cl. 47/TrkB | BD Bioscience | Weak staining | -- | -- |
| TrkB cat. AB5372 | Millipore | Weak staining | -- | -- |
| LRP5/6 cl. 1A12 | Millipore | Weak staining | -- | -- |
| ISLR-2 cat. AF4650 | R&D Systems | Weak staining | -- | -- |
| CD54 cl. KAT1 | eBioscience | Weak staining | -- | -- |
| Glast cat. AF6048 | R&D Systems | Weak staining | -- | -- |
| c-Kit cl. 3C1 | Southern Biotech | Weak staining | -- | -- |
| MAA lectin | EY Labs | Weak staining (uniform) | -- | -- |
| CD61 cl. 2C9.G3 | eBioscience | No staining | -- | -- |
| ESAM cl. 1G8 | eBioscience | No staining | -- | -- |
| OMGP cl. AF1674 | R&D Systems | No staining | -- | -- |
| GS-I-B4 lectin | eBioscience | No staining | -- | -- |
| CD48 cl. HM48-1 | eBioscience | No staining | -- | -- |
| Tag-1 cl. 4D7 | DSHB | No staining | -- | -- |
| CD56 cl. 12F11 | BD Bioscience | No staining | -- | -- |
| CD5 cl. 53-7.3 | eBioscience | No staining | -- | -- |
| CD106 cl. 429 | eBioscience | No staining | -- | -- |
| CD322 cl. CRAM-19 H36 | AbD Serotec | No staining | -- | -- |
| CD322 cl. CRAM-18 F26 | AbD Serotec | No staining | -- | -- |
| CD147 cl. RL73 | eBioscience | No staining | -- | -- |
| CD73 cl. TY/23 | BD Bioscience | No staining | -- | -- |
| CD140b/PDGFRb cl. APB5 | eBioscience | No staining | -- | -- |
| Laminin gamma 1 cat. sc-6019 | Santa Cruz | No staining | -- | -- |
| CD120b cl. TR75-89 | BD Bioscience | No staining | -- | -- |
| CD120a cl. 55R-286 | BD Bioscience | No staining | -- | -- |
| CD178 cl. MFL3 | BD Bioscience | No staining | -- | -- |
| CD42d cl. 1C2 | BD Bioscience | No staining | -- | -- |
| Dll1 Genentech | Genentech | No staining | -- | -- |
| p75 cat. NE1024 | Millipore (Calbiochem) | No staining | -- | -- |
| p75 cat. GR10 | Oncogene | No staining | -- | -- |
| hBMPR1a | unknown | No staining | -- | -- |
| h/mBMPR1b | unknown | No staining | -- | -- |
| CD54 cl.RR1/1 | eBioscience | No staining | -- | -- |
| Alcam cl. F84.1 | Santa Cruz | No staining | -- | -- |
| CD49c cl. P1B5 | eBioscience | No staining | -- | -- |
| CD247 cl. 8D3 | BD Bioscience | No staining | -- | -- |
| IFNAR1 cl. MAR1-5A3 | BD Bioscience | No staining | -- | -- |
| Fibronectin cat. 610077 | BD Bioscience | No staining | -- | -- |
| Shh cl. 5E1 | DSHB | No staining | -- | -- |
| Fibronectin cat. MAB88904 | Millipore | No staining | -- | -- |
| Collagen I cat. ab6308 | Abcam | No staining | -- | -- |
| GRL-2 | DSHB | No staining | -- | -- |
| Tag-1 cl. 3.1C12 | DSHB | No staining | -- | -- |
| HCS-1 | DSHB | No staining | -- | -- |
| CD157 cl. BP-3 | BD Bioscience | No staining | -- | -- |
| GRL-1 | DSHB | No staining | -- | -- |
| 3CB2 | DSHB | No staining | -- | -- |
| RC2 | DSHB | No staining | -- | -- |
| FE-A5 | DSHB | No staining | -- | -- |
| FE-C6 | DSHB | No staining | -- | -- |
| FE-J1 | DSHB | No staining | -- | -- |
| Laminin receptor cl. MLuC5 | Abcam | No staining | -- | -- |
| N-cadherin cat. 13116 | Cell Signaling | No staining | -- | -- |
| CD10 cat. NBP2-15771 | Novus | No staining | -- | -- |
| N-cadherin cat. 18571 | IBL | No staining | -- | -- |
| CD193 cl. J073E5 | Biolegend | No staining | -- | -- |
| Collagen IV cat. ab52235 | Abcam | No staining | -- | -- |
| Laminin 1+2 cat. Ab7463 | Abcam | No staining | -- | -- |
| Laminin cat. AB2034 | Millipore | No staining | -- | -- |
| Collagen IV cat. AB756P | Millipore | No staining | -- | -- |
| ADAM23 cat. sc-50482 | Santa Cruz | No staining | -- | -- |
| N-cadherin cl. MNCD2 | DSHB | No staining | -- | -- |
| CD210 cl. 1B1.3a | BD Bioscience | No staining | -- | -- |
| CD38 cl. 90 | eBioscience | No staining | -- | -- |
| CD40 cl. 1C10 | eBioscience | No staining | -- | -- |
| BP1 cl. FG35.4 | eBioscience | No staining | -- | -- |
| BP1 cl. 6C3 | eBioscience | No staining | -- | -- |
| CD19 cl. 1D3 | eBioscience | No staining | -- | -- |
| Laminin B2 cl. A5 05-206 | Millipore | No staining | -- | -- |
| Laminin B1 cl. LT3 | Thermo Scientific | No staining | -- | -- |
| CD107b cl. M3/84 | BD Bioscience | No staining | -- | -- |
| CD18 cl. M18/2 | eBioscience | No staining | -- | -- |
| TROMA-1 | DSHB | No staining | -- | -- |
| CD52 cl. H186 | Biolegend | No staining | -- | -- |
| MHC II cl. NIMR-4 | eBioscience | No staining | -- | -- |
| SSEA-3 | DSHB | No staining | -- | -- |
| CD107b cl. ABL-93 | eBioscience | No staining | -- | -- |
| MD1 cl. MD14 | BD Bioscience | No staining | -- | -- |
| Ly51 cl. 6C3 | Biolegend | No staining | -- | -- |
| CD196 cl. 140706 | BD Bioscience | No staining | -- | -- |
| CD193 cl. 83103 | BD Bioscience | No staining | -- | -- |
| CD201 cl. 1560 | eBioscience | No staining | -- | -- |
| CD4 cl. GK1.5 | eBioscience | No staining | -- | -- |
| CD229 cl. Ly9ab3 | Biolegend | No staining | -- | -- |
| CD30 cl. mCD30.1 | eBioscience | No staining | -- | -- |
| CD43 cl. W3/13 | Biolegend | No staining | -- | -- |
| TIM1 cl. RMT1-4 | eBioscience | No staining | -- | -- |
| TIM2 cl. RMT2-1 | eBioscience | No staining | -- | -- |
| Sema4a cl. KL-1 | eBioscience | No staining | -- | -- |
| CD80 cl. 16-10A1 | Caltag | No staining | -- | -- |
| CD122 cl. TM-beta-1 | Caltag | No staining | -- | -- |
| CD104 cl. 346-11A | Biolegend | No staining | -- | -- |
| CD266 cl. ITEM-4 | Biolegend | No staining | -- | -- |
| CD254 cl. IK22/5 | Biolegend | No staining | -- | -- |
| CD80 cl. 16-10A1 | BD Bioscience | No staining | -- | -- |
| CD278 cl. 7E.17G9 | BD Bioscience | No staining | -- | -- |
| SBA lectin | EY Labs | No staining | -- | -- |
| AAA lectin | EY Labs | No staining | -- | -- |
| VEA lectin | EY Labs | No staining | -- | -- |
| VEGFR3 cat. AF743 | R&D Systems | No staining | -- | -- |
| CD144 cl. BV13 | eBioscience | No staining | -- | -- |
| CD62E cl. 10E9.6 | BD Bioscience | No staining | -- | -- |
| CD109 cat. MAB4385 | R&D Systems | No staining | -- | -- |
| CD146 cl. P1H12 | eBioscience | No staining | -- | -- |
| CD72bc cl. JY/93 | BD Bioscience | No staining | -- | -- |
| CD102 cl. 3C4 | eBioscience | No staining | -- | -- |
| CD253 cl. N2B2 | eBioscience | No staining | -- | -- |
| CD62P cl. Pse1.KO2.3 | eBioscience | No staining | -- | -- |
| CD134 cl. OX-86 | eBioscience | No staining | -- | -- |
| CD126 cl. D7715A7 | eBioscience | No staining | -- | -- |
| IaIe cl. M5/114.15.2 | Biolegend | No staining | -- | -- |
| CD103 cl. M290 | BD Bioscience | No staining | -- | -- |
| CD209a cl. 5H10 | BD Bioscience | No staining | -- | -- |
| IBL-9/2 | unknown | No staining | -- | -- |
| IBL-7/1 | unknown | No staining | -- | -- |
| PNA lectin | EY Labs | No staining | -- | -- |
| CD11a cl. M17/4 | eBioscience | No staining | -- | -- |
| CD115 cl. AFS98 | eBioscience | No staining | -- | -- |
| CD25 cl. PC61.5 | eBioscience | No staining | -- | -- |
| c-Met cl. eBioclone 4 | eBioscience | No staining | -- | -- |
| Ly49g2 cl. 4D11 | eBioscience | No staining | -- | -- |
| Itgb7 cl. FIB504 | eBioscience | No staining | -- | -- |
| CD72abd cl. J4-117 | eBioscience | No staining | -- | -- |
| CD269 cl. 19F2 | BD Bioscience | No staining | -- | -- |
| BS1 lectin cat. L3759 | Sigma | No staining | -- | -- |
| c-Kit cl. 3C11 | Santa Cruz | No staining | -- | -- |
| CD123 cl. 5B11 | eBioscience | No staining | -- | -- |
| CD26 cl. H194-112 | Biolegend | No staining | -- | -- |
| H2D-b cl. KH95 | Biolegend | No staining | -- | -- |
| CD94 cl. 18d3 | eBioscience | No staining | -- | -- |
| CD44 cl. 5035-41.D | Abcam | No staining | -- | -- |
| UEA-1 and 2 lectin | EY Labs | No staining | -- | -- |
| GS-1 lectin | EY Labs | No staining | -- | -- |
| CD49b cl. DX5 | eBioscience | No staining | -- | -- |
| CD34 cl. RAM34 | eBioscience | No staining | -- | -- |
| CD36 cl. No72-1 | eBioscience | No staining | -- | -- |
| CD28 cl. 37.51 | eBioscience | No staining | -- | -- |
| CD14 cl. Sa2-8 | eBioscience | No staining | -- | -- |
| CD223 cl. C9B7W | eBioscience | No staining | -- | -- |
| CD1d cl. 1B1 | eBioscience | No staining | -- | -- |
| CD22 cl. OX-97 | Biolegend | No staining | -- | -- |
| CD23 cl. B3B4 | eBioscience | No staining | -- | -- |
| CD70 cl. FR70 | eBioscience | No staining | -- | -- |
| CD80 ebiosci cl. 16-10A1 | eBioscience | No staining | -- | -- |
| CD83 cl. HB15e | eBioscience | No staining | -- | -- |
| CD103 cl. 2E7 | eBioscience | No staining | -- | -- |
| CD122 cl. TM-beta1 | eBioscience | No staining | -- | -- |
| CD137 cl. 17B5 | eBioscience | No staining | -- | -- |
| CD152 ebiosci cl. UC10-4B9 | eBioscience | No staining | -- | -- |
| CD154 cl. MR1 | eBioscience | No staining | -- | -- |
| CD195 cl. 7A4 | eBioscience | No staining | -- | -- |
| CD197 cl. 4B12 | eBioscience | No staining | -- | -- |
| TIM3 cl. 8B2C12 | eBioscience | No staining | -- | -- |
| B7H4 cl. 188 | eBioscience | No staining | -- | -- |
| GITRL cl. YGL-386 | eBioscience | No staining | -- | -- |
| Il21R cl. 4A9 | BD Bioscience | No staining | -- | -- |
| NKG2D cl. A10 | eBioscience | No staining | -- | -- |
| F4/80 cl. BM8 | eBioscience | No staining | -- | -- |
| Ly49a/d cl. 12A8 | eBioscience | No staining | -- | -- |
| CD150 cl. TC15-12F12.2 | Biolegend | No staining | -- | -- |
| CD56 cl. CMSSB | eBioscience | No staining | -- | -- |
| CD11c cl. N418 | Biolegend | No staining | -- | -- |
| Dll1 cl. HMD1-5 | eBioscience | No staining | -- | -- |
| CD68 cl. FA-11 | Biolegend | No staining | -- | -- |
| CD137L cl. TKS-1 | eBioscience | No staining | -- | -- |
| CD276 cl. M3.2D7 | eBioscience | No staining | -- | -- |
| CD88 cl. 20/70 | Biolegend | No staining | -- | -- |
| CD131 cl. JORO50 | BD Bioscience | No staining | -- | -- |
| CD84 cl. mCD84.7 | Biolegend | No staining | -- | -- |
| Ly108 cl. 330-AJ | Biolegend | No staining | -- | -- |
| CD127 cl. A7R34 | Biolegend | No staining | -- | -- |
| CD62L cl. MEL-14 | Biolegend | No staining | -- | -- |
| CD27 cl. LG.3A10 | Biolegend | No staining | -- | -- |
| CD180 cl. RP1/4 | Biolegend | No staining | -- | -- |
| CD69 cl. H1.2F3 | Biolegend | No staining | -- | -- |
| CD86 cl. GL-1 | Biolegend | No staining | -- | -- |
| Thy1-2 cl. 30-H12 | eBioscience | No staining | -- | -- |
| IL4 cl. 11B11 | Biolegend | No staining | -- | -- |
| H2D-d cl. 34-2-12 | Biolegend | No staining | -- | -- |
| CD135 cl. A2F10 | Biolegend | No staining | -- | -- |
| Gp49r cl. H1.1 | Biolegend | No staining | -- | -- |
| Flk1 cl. Avas12a1 | eBioscience | No staining | -- | -- |
| CD95 cl. 15A7 | eBioscience | No staining | -- | -- |
| CD41 cl. MWReg30 | Biolegend | No staining | -- | -- |
| Notch1 cl. MN1a | eBioscience | No staining | -- | -- |
| CD70 cl. FR70 | eBioscience | No staining | -- | -- |
| Cxcr5 cl. SPRCL5 | eBioscience | No staining | -- | -- |
| CD13 cl. R3-242 | BD Bioscience | No staining | -- | -- |
| IgDb cat. 553511 | BD Bioscience | No staining | -- | -- |
| FcgII cat. 01245B | BD Bioscience | No staining | -- | -- |
| CD150 cl. mShad150 | eBioscience | No staining | -- | -- |
| Ly108 cl. 13G3-19D | eBioscience | No staining | -- | -- |
| CD1d cl. 3C11 | BD Bioscience | No staining | -- | -- |
| CD2 cl. RM2-5 | eBioscience | No staining | -- | -- |
| CD3e cl. 145-2C11 | eBioscience | No staining | -- | -- |
| CD8a cl. 53-6.7 | eBioscience | No staining | -- | -- |
| Gr-1 cl. RB6-8C5 | eBioscience | No staining | -- | -- |
| TNFalpha cl. MP6-XT22 | eBioscience | No staining | -- | -- |
| CD11b/Mac-1 cl. M1/70 | eBioscience | No staining | -- | -- |
| CD153 cl. RM153 | BD Bioscience | No staining | -- | -- |
| GITR cl. DTA-1 | eBioscience | No staining | -- | -- |
| CD244 cl. ebio244F4 | eBioscience | No staining | -- | -- |
| CD244 cl. m2B4 | Biolegend | No staining | -- | -- |
| Syndecan-1 cl. 281-2 | BD Bioscience | No staining | -- | -- |
| CD93 cl. AA4.1 | eBioscience | No staining | -- | -- |
| CD28 cl. 37.51.1 | Caltag | No staining | -- | -- |
| Fitc-PdgfBB | Morrison lab | No staining | -- | -- |
| CD45R cl. RA3-6B2 | eBioscience | No staining | -- | -- |
| 9BA12 | DSHB | No staining | -- | -- |
| KH10 | DSHB | No staining | -- | -- |
| LA4 | DSHB | No staining | -- | -- |
| LAM-B | DSHB | No staining | -- | -- |
| Tnfrsf19 cat. AF723 | R&D Systems | No staining | -- | -- |
| Sema6C cat. AF2108 | R&D Systems | No staining | -- | -- |
| Hepacam cat. AF4108 | R&D Systems | No staining | -- | -- |
| CD22.2 cl. Cy34.1 | BD Bioscience | No staining | -- | -- |
| Pancortin cl. K96/7 | NeuroMab | No staining | -- | -- |
| ADAM22 cl. N57/2 | NeuroMab | No staining | -- | -- |
| LRP4 cl. N207/27 | NeuroMab | No staining | -- | -- |
| Neurexin-1-Beta cl. N170A/1 | NeuroMab | No staining | -- | -- |
| Unc5D cat. 20241-1-AP | ProteinTech | No staining | -- | -- |
| LHR cat. NLS1436 | Novus | No staining | -- | -- |
| Igf1R cat. AF-305-NA | R&D Systems | No staining | -- | -- |
| CD37 cl. IPO-24 | Novus | No staining | -- | -- |
| Caspr2 cl. K67/25 | NeuroMab | No staining | -- | -- |
| SynCAM1 cl. L45/30 | NeuroMab | No staining | -- | -- |
| Lrrtm2 cl. ab169857 | Abcam | No staining | -- | -- |
| Pmp22 cat. ABIN484001 | anibodies online | No staining | -- | -- |
| MZ15 | DSHB | No staining | -- | -- |
| GAD-6 | DSHB | No staining | -- | -- |
| L6 tumor antigen cl. D1-D2 | Millipore | No staining | -- | -- |
| N-cadherin cl. 3C4 | BD Bioscience | No staining | -- | -- |
| TrkC cl. 2B7 | Novus | No staining | -- | -- |
| TrkC cat. Ab72120 | Abcam | No staining | -- | -- |
| TrkB cl. 6B10 | Novus | No staining | -- | -- |
| Ghr cat. bs-0654R | Bioss | No staining | -- | -- |
| Ghr cat. sc-57161 PE | Santa Cruz | No staining | -- | -- |
| GSII lectin | EY Labs | No staining | -- | -- |
| SJA lectin | EY Labs | No staining | -- | -- |
| EEA lectin | EY Labs | No staining | -- | -- |
| Cmklr1 cl. BZ194 | eBioscience | No staining | -- | -- |
| Siglec H cl. eBio440c | eBioscience | No staining | -- | -- |
| Notch3 cl. HMN3-133 | eBioscience | No staining | -- | -- |
| CD49d cl. R1-2 | eBioscience | No staining | -- | -- |
| CD49d cl. 9C10(MFR4.B) | Biolegend | No staining | -- | -- |
| Cxcr7 cl. 8F11-M16 | Biolegend | No staining | -- | -- |
| VVL lectin | Vector Labs | No staining | -- | -- |
| Jagged-1 cl. HMJ1-29 | eBioscience | No staining | -- | -- |
| SSEA-4 | DSHB | No staining | -- | -- |
| Notch2 cl. 16F11 | eBioscience | No staining | -- | -- |
| CD274 cl. MIH5 | eBioscience | No staining | -- | -- |
| CD138 cl. 281-2 | BD Bioscience | No staining | -- | -- |
| SDF1 cat. MAB350 | R&D Systems | No staining | -- | -- |
| CD326 cl. G8.8 | BD Bioscience | No staining | -- | -- |
| CD45RB cl. C363.16A | eBioscience | No staining | -- | -- |
| NCAM cl. 5B8 | BD Bioscience | No staining | -- | -- |
| HGFR cat. AF527 | R&D Systems | No staining | -- | -- |
| p75 cat. MAB5592 | Millipore | No staining | -- | -- |
| 1B11 GPI-linked Neurocan receptor | DSHB | No staining | -- | -- |
| 7A2 GPI-linked Neurocan receptor | DSHB | No staining | -- | -- |
| Gpc2 cat. AF2355 | R&D Systems | No staining | -- | -- |
| Itga8 cat. AF4076 | R&D Systems | No staining | -- | -- |
| CD43 cl. eBioR2/60 | eBioscience | No staining | -- | -- |
| CD209 cl. 5H10 | eBioscience | No staining | -- | -- |
| CD45RC cl. GL24 | eBioscience | No staining | -- | -- |
| 488-bFGF | Morrison lab | No staining | -- | -- |
| ECL lectin | Vector Labs | No staining | -- | -- |
| CD44 cl. IM7 | BD Bioscience | No staining | -- | -- |
| Itgb5 cl. KN52 | eBioscience | No staining | -- | -- |
| alpha-Dystroglycan cat. sc-53987 | Santa Cruz | No staining | -- | -- |

| **Supplementary File 1B: Candidate growth factors and medium supplements screened for the ability to stimulate colony formation by pre-GEPCOT cells.** | | | | | | |
| --- | --- | --- | --- | --- | --- | --- |
|  |  |  | **Percent of colonies formed in control cultures** | | | |
|  |  |  | **(* p < 0.05, *** p < 0.001)** | | | |
| **Culture additive** | **Source** | **Concentration** | **Effect on neurosphere formation by unfractionated SVZ cells** | **Effect on neurosphere formation by unfractionated TMZ-treated SVZ cells** | **Effect on adherent colony formation by unfractionated SVZ cells** | **Effect on adherent colony formation by unfractionated TMZ-treated SVZ cells** |
| Smoothened Agonist (SAG) | EMD Millipore | 500 nM | 135 | 200 | 72 | 154 |
| Shh | Miltenyi Biotec | 20 ng/ml | 134 | 74 | 129 | 63 |
| LIF | eBioscience | 20 ng/ml | 112 | 155 | 97 | 115 |
| Noggin | R&D Systems | 25 ng/ml | 119 | 274 | 110 | 40 |
| Forskolin | Sigma | 10 μM | 130 | 174 | 95 | 70 |
| Prokineticin-1 | Peprotech | 20 ng/ml | -- | -- | 123 | 67 |
| Prokineticin-2 | Peprotech | 20 ng/ml | -- | -- | 62 | 67 |
| CNTF | R&D Systems | 20 ng/ml | 90 | 74 | 108 | 100 |
| BDNF | R&D Systems | 20 ng/ml | 82 | 37 | 89 | 94 |
| Sphingosine-1-phosphate | Sigma | 100 nM | -- | -- | 67 | 79 |
|  |  | 100 nM | 100 nM: 117 | 100 nM: 12 | 100 nM: 141 | 100 nM: 48 |
| Dimethyl PGE2 | Tocris | 1 μM | 1 μM: 76 | 1 μM: 0 |  |  |
|  |  | 10 μM | 10 μM: 0 *** | 10 μM: 0 |  |  |
| Jag1-Fc | Life Technologies | 20 ng/ml | 109 | 37 | 40 | 78 |
| Neuropeptide Y | Sigma | 40 ng/ml | 98 | 111 | -- | -- |
| IGF2 | R&D Systems | 20 ng/ml | 94 | 37 | -- | -- |
| PDGF-AA | eBioscience | 20 ng/ml | 124 | 211 | 91 | 38 |
| PDGF-BB | R&D Systems | 20 ng/ml | 109 | 37 | -- | -- |
| GDNF | R&D Systems | 20 ng/ml | 105 | 74 | 138 | 100 |
| NGF-beta | Sigma | 50 ng/ml | 102 | 111 | 99 | 53 |
| FBS | Life Technologies | 1% | -- | -- | 89 | 181 |
| NT3 | R&D Systems | 20 ng/ml | 109 | 111 | -- | -- |
| BMP7 | R&D Systems | 20 ng/ml | 17 *** | 0 | -- | -- |
| BMP4 | Sigma | 20 ng/ml | 12 *** | 0 | -- | -- |
| BMPER | R&D Systems | 100 ng/ml | 110 | 0 | -- | -- |
| Htra1 | Origene | 50 ng/ml | 106 | 147 | -- | -- |
| Nrg1 | R&D Systems | 20 ng/ml | 103 | 74 | -- | -- |
| Follistatin | Origene | 20 ng/ml | 89 | 147 | -- | -- |
| Follistatin-like | Origene | 20 ng/ml | 85 * | 184 | -- | -- |
| Pleiotrophin | R&D Systems | 20 ng/ml | 91 | 0 | -- | -- |
| Midkine | Sigma | 20 ng/ml | 105 | 111 | -- | -- |
| HGF | Miltenyi Biotec | 20 ng/ml | 112 | 92 | -- | -- |
| Caffeine | Sigma | 1 mM | 84 * | 0 | -- | -- |
| TGF-beta1 | R&D Systems | 20 ng/ml | 86 | 74 | -- | -- |
| ECGS | Biomedical Technologies Inc. | 100 μg/ml | -- | -- | 115 | 100 |
| SCF | R&D Systems | 50 ng/ml | 131 | 165 | 80 | 85 |
| Wnt3a | R&D Systems | 20 ng/ml | 96 | 0 | 130 | 67 |
| Sheep serum | Sigma | 1% | -- | -- | 57 | 86 |
